# Effect of Age and Sodium Alendronate on Femoral Fracture Repair: Biochemical and Biomechanical Study in Rats

**DOI:** 10.3389/fcell.2021.558285

**Published:** 2021-05-05

**Authors:** Luana Mordask Bonetto, Paola Fernanda Cotait de Lucas Corso, Gabrielle Grosko Kuchar, Jennifer Tsi Gerber, Leonardo Fernandes Cunha, Mohammed Elsalanty, João Cesar Zielak, Carla Castiglia Gonzaga, Rafaela Scariot

**Affiliations:** ^1^Department of Dentistry, Positivo University, Curitiba, Brazil; ^2^Department of Medical Anatomical Science, Western University of Health Sciences, Pomona, CA, Unioted States; ^3^Department of Dentistry, Federal University of Parana, Curitiba, Brazil

**Keywords:** fracture, femur, bisphosphonates, rats, bone regeneration

## Abstract

**Background:**

Bisphosphonates are drugs widely used to reduce bone resorption, increase bone mineral density and control age-related bone loss. Although there are studies reporting differences in bone structure between young and old adults, it is still difficult to predict changes related to bone aging. The aim of this study was to evaluate the effect of age and sodium alendronate on bone repair of femoral fractures in rats.

**Methods:**

*Wistar* rats (*n* = 40) were allocated into groups: O (control old-rats), Y (control young-rats), OA (alendronate old-rats) and YA (alendronate young-rats). All animals underwent linear fracture surgery followed by fixation. Groups OA and YA received 1 mg/kg alendronate three times a week until euthanasia. Biochemical analysis of calcium and alkaline phosphatase was done. After euthanasia, femurs were evaluated in relation to cross-section and flexural strength, with three-point bending test. Data were submitted to statistical analysis with significance level of 0.05.

**Results:**

There was no difference in calcium and alkaline phosphatase levels (*p* > 0.05). Young animals presented lower cross-section than older animals (*p* < 0.05). Only fractured side, young animals presented major flexural strength than older animals (*p* < 0.05). There was no difference between the animals that used or not alendronate in relation to cross-section and flexural strength (*p* > 0.05). When compared fractured and non-fractured femurs, major cross-section on fractured side was observed (*p* < 0.05). Flexural strength presented higher values in femurs on non-fractured side (*p* < 0.05). There was correlation of weight and cross-section (*R* = +0.91) and weight with flexural strength of fractured and non-fractured side, respectively (*R* = −0.97 and −0.71).

**Conclusion:**

In short, there was no difference of calcium and alkaline phosphatase during the bone repair process. Age has influence in cross-section and flexural strength. Alendronate showed no association with these factors.

## Introduction

Mineralized bone matrix is an important reservoir of calcium and phosphate ions (Redmond et al., 2014). Alkaline phosphatase is produced in high concentration during bone formation and is, therefore, a good indicator of bone regeneration activity (Christenson, 1997). During bone regeneration after fracture, hematopoietic precursor cells migrate to the area and differentiate into osteoclasts to digest the necrotic bone, while mesenchymal stromal cells differentiate into bone-forming osteoblasts. During the remodeling of the bone callus, catabolic and anabolic activities interact, a balance that can be modulated by different molecules (Bosemark et al., 2014). The process of bone healing and remodeling are known to occur more rapidly in young individuals (Wendeberg, 1961). Although there are studies reporting differences in bone structure between young and old adults (Akkus et al., 2004), it is still difficult to predict changes related to bone aging. Bone aging is associated with a decrease in collagen content and bone mineral density (Bailey et al., 1999). Consequently, there is a decrease in elastic capacity of bone with age and a greater difficulty in bone healing.

Several molecules have shown potential to stimulate osteogenic activity or reduce bone resorption *in vitro* and *in vivo* (Greiner et al., 2008). Bisphosphonates are examples of drugs that increase bone mineral density and control age-related bone loss (Naruse et al., 2015). There is a decrease in the osteoclastic activity, without direct effect on bone formation (Greiner et al., 2008). Bisphosphonates bind to hydroxyapatite and can remain in bone matrix for years (Silva et al., 2010). Studies indicated that a single systemic dose of alendronate significantly increased the volume of bone callus during fracture repair and increased the bone mechanical strength. When administered in the perioperative period, it induced 30% greater mechanical resistance, compared to controls. Administration of the systemic dose within one or 2 weeks after fracture increased the mechanical strength in the regenerate by 44 and 50%, respectively, confirming the effect of the drug on bone healing (Amanat et al., 2007).

The objective of this study was to evaluate the effect of age and systemic application of alendronate on the fracture resistance of bone regenerate after controlled femoral fracture. We hypothesized that fracture resistance of the regenerate will decrease with age and that alendronate treatment would, at least partially, correct such negative effect.

## Materials and Methods

### Ethical Aspects

The research was carried out in the Vivarium and in the Research Laboratory at Universidade Positivo, after approval in the Ethics Committee on the Use of Animals (ECUA 393). The animals experiments were conducted in accordance to the ARRIVE guidelines, as well as the National Institutes of Health guide for the care and use of Laboratory animals.

### Experimental Design

In this study, forty male Wistar rats (Rattus norvegicus) were used: 20 middle-aged rats with 1 year and 5 months of age, with approximately 500 grams and 20 young rats with 5 months of age weighing approximately 200 grams (Andreollo et al., 2012). The rats were divided into four groups: Group O (control) and Group OA (systemic alendronate application), using rats with 1 year and 5 months of age, Group Y (control) and Group YA (systemic alendronate application), using the rats with 5 months old. The dosage used of alendronate for group OA and YA was 1 mg/kg, with three times a week applications, in intraperitoneal region, until euthanasia, starting immediately after the fracture induction. The dose of choice is based on preliminary works of our team (de Oliveira et al., 2019). Subcutaneous applications were performed on the dorsal region of the rats, introducing the needle parallel to the fold of body tissue formed by digital pressure.

Considering the importance of the regulation of calcium and alkaline phosphatase for bone metabolism, blood samples were taken at three different times for biochemical analysis. From the total of 40 animals, 20% had material collected and distributed equally among the groups.

Throughout the experiment, the ambient conditions of light, temperature and humidity of the rooms were controlled in a digital panel in order to maintain the photoperiod of 12 h, with the temperature ranging from 18 to 22°C and relative humidity of 65%.

### Surgical Procedure

The rats were sedated with isoflurane 3% (Cristália, Itapira, SP, Brazil), then anesthetized with 10% ketamine hydrochloride (75 mg/kg) (Vetbrands, Paulínia, SP, Brazil) with 2% (10 mg/kg) xylazine hydrochloride (Vetbrands, Paulínia, SP, Brazil), by intraperitoneal injection.

**FIGURE 1:**
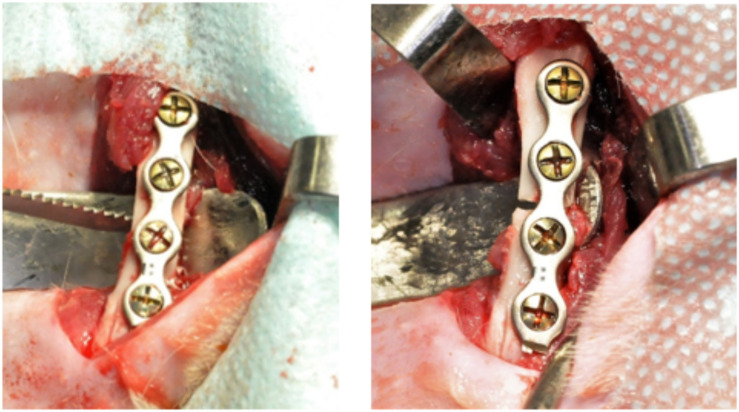
4-hole titanium plate of 2.0 mm system with four screws of 2.0 mm and fracture performed using a reciprocating saw.

After the anesthesia, the rats were positioned in left lateral decubitus position; and the right femur trichotomy was performed with vigorous antisepsis using iodopovidone. A straight incision along the axis of the femur was made, approximately 3 cm long, with a 15C scalpel blade. With blunt scissors, the tissues were dissected into muscular planes. Next, the periosteum was incised with a scalpel and elevated with a delicate syndesmotom, thus accessing the cortical surface of the femur. A 4-hole titanium plate of 2.0 mm system with four screws of 2.0 mm system being 5 mm long, was adapted to the bone surface prior to osteotomy in order to avoid the poor positioning of the bone segments. The fracture was then performed using a reciprocating saw (NSK, Shinagawa, Tokyo, Japan) under constant irrigation. The stability of titanium plate and screws, hemostasis and abundant wound washing with saline solution were reviewed (Figure 1). The suture was performed in planes with isolated stitches using Vycril 4–0 (Ethicon, Johnson & Johnson, São José dos Campos, SP, Brazil) for the muscles, and nylon 4–0 (Ethicon, Johnson & Johnson, São José dos Campos, SP, Brazil) for the skin.

To avoid postoperative infections, we used 10 mg/kg of broad-spectrum antibiotic (Kinolox 2.5%, World Animal Laboratory, Pindamonhangaba—Brazil) intraperitoneally every 24 h for 14 days. ketoprofen 50 mg/ml, at 5 mg/kg, was given intraperitoneally every 24 h for 5 days to control post-operative inflammation. For analgesia, tramadol hydrochloride 100 mg/2 ml was given intraperitoneally at the end of surgery and was maintained for 5 days, with a dosage of 7 mg/kg (União Química, Jabaquara, SP, Brazil). For gastric protection, ranitidine 15 mg/ml with a dose of 5 mg/kg, was orally administrated, every 24 h for 3 days.

In the postoperative period the animals were kept in their own cages and fed daily with heavy ration for appetite control (100 mg/day) and water at will. The surgical wound was cleaned with sterile gauze and iodopovidone, once a day, for 5 days. At the seventh day, the animals were evaluated for the presence of suture and when present was removed.

Seven days after the surgical procedure, the rats were sedated with isoflurane in order to perform the digital radiographs (Kodak x-ray sensor, Rochester, New York, United States), which were used to evaluate the positioning of the bone stumps as well as the plates and screws. After the rats were euthanized and the plates and screws removed, another digital radiograph was performed to observe the bone repair in the fracture site. Based on this exam (complete bone repair), femurs were submitted to mechanical tests.

### Medication Applications

In the OA and YA groups, immediately after surgery, the application of alendronate was initiated until the day of euthanasia. The drug was administered three times a week with 1 mg/kg dosage. Groups O and Y, at the same time, received applications of saline solution at 0.9%.

### Euthanasia

The animals were euthanized 90 days after the procedure, with inhalation of 3% isoflurane until complete absence of vital signs and then the fractured and non-fractured femurs were removed, clinically evaluated and stored in bottles.

### Biochemical Analysis

Blood samples were collected (1 ml) by cardiac puncture, under sedation with isoflurane in three different times: after the fracture induction, after 45 days and after 90 days from the surgical procedure. Samples were sent to biochemical analysis for levels of calcium and alkaline phosphatase (VP veterinary biochemical laboratory—Curitiba—Paraná—Brazil) of groups O, Y, OA, and YA.

### Mechanical Test

Three-point bending flexural tests were performed in a universal testing machine (DL2000, EMIC, São José dos Pinhais, PR, Brazil) at a crosshead speed of 1 mm/min. The distance between the supports was 16 mm. Before testing, specimens’ dimensions were determined using a digital caliper with 0.01 mm accuracy. Cross-section (in mm2) was calculated using the formula CS = hb, where h corresponds to height (in mm) and b corresponds to width (in mm). Flexural strength (S, in MPa) was calculated using the following formula: S = 3Fl/2bh2, where F is the failure load (in Newtons), l is the distance between the supports, b is the width and h is the height of the specimen (all in mm).

### Statistical Analysis

The variables were subjected to statistical analysis. To evaluate the association of calcium and alkaline phosphatase between groups was used the One-Way ANOVA test and to evaluate the association between serum dosages of the same group between the times was used the One-Way ANOVA of repeated measures, using the mean and standard deviation to represent the sample. To evaluate the association between cross-section of the femurs and flexural strength with the factors age and group, as well as their interactions, was used the Two-Way ANOVA test with Tukey’s Posttest. To compare the cross-section and flexural strength between fractured and non-fractured femurs in animals treated with alendronate or not, was used the paired student *T* test. To evaluate the correlation of weight with cross-section and the weight with flexural strength, in femurs with and without fracture, the Pearson’s correlation test was used. The statistical analysis was performed using the Statistical Packager Social Science program (SPSS, version 21.0, SPSS inc., Chicago, IL, United States), with a significance level of 0.05.

## Results

Tables 1, 2 outline the calcium and alkaline phosphatase levels at different time points in different groups. There were no significant differences in serum calcium and alkaline phosphatase levels evaluated at the same time and over time between the groups (*p* > 0.05).

**TABLE 1:** Serum levels of calcium at different times.

Age	Alendronate	Calcium Pre-operative Mean ± SD	Calcium 45 days Mean ± SD	Calcium 90 days Mean ± SD	*P* Value Time
Middle-age	No (O)	9.40 ± 0.0	10.20 ± 0.20	14.00 ± 1.50	0.156
	Yes (OA)	10.23 ± 0.42	9.66 ± 0.75	9.73 ± 0.75	0.932
Young	No (Y)	10.55 ± 0.35	10.50 ± 0.30	9.70 ± 0.50	0.500
	Yes (YA)	11.15 ± 0.65	10.80 ± 0.90	9.70 ± 0.30	0.395
*P* Value Group		0.179	0.669	0.051	

*One-Way ANOVA for evaluation between groups and One-Way ANOVA repeated measures for evaluation between times, with significance level of 0.05.*

**TABLE 2:** Serum levels of alkaline phosphatase in the groups at different times.

Age	Alendronate	Alkaline phosphatase Pre-operative Mean ± SD	Alkaline phosphatase 45 days Mean ± SD	Alkaline phosphatase 90 days Mean ± SD	*P* Value Time
Middle-age	No (O)	116.15 ± 9.35	111.15 ± 22.45	122.80 ± 5.80	0.901
	Yes (OA)	125.03 ± 39.00	110.20 ± 15.17	65.03 ± 5.57	0.525
Young	No (Y)	171.70 ± 36.60	157.15 ± 6.15	131.20 ± 30.10	0.791
	Yes (YA)	225.85 ± 47.05	93.00 ± 10.10	119.20 ± 20.80	0.173
*P* Value Group		0.290	0.144	0.082	

*One-Way ANOVA for evaluation between groups and One-Way ANOVA repeated measures for evaluation between times, with significance level of 0.05.*

Table 3 demonstrate the cross-sectional area and flexural strength of middle aged and young rats with and without alendronate applications. Regarding the cross-section on the fracture side, middle aged animals had higher values than young animals (53.52 versus 32.42 mm^2^, respectively; *p* < 0.05). Regarding the effect of alendronate treatment, there was no difference between treated (47.50 mm^2^) and untreated animals (42.32 mm^2^). The variables alendronate and age/alendronate did not present statistically significant difference (*p* > 0.05).

**TABLE 3:** Evaluation for cross-section and flexural strength related to age and presence or not of alendronate.

Age	Alendronate	Cross-section (mm2) fracture Mean ± SD	Cross-section (mm2) no fracture Mean ± SD	Flexural strength (MPa) fracture Mean ± SD	Flexural strength (MPa) no fracture Mean ± SD
Middle-age	No (O)	51.70 ± 22.37^Aa^	20.30 ± 2.32 ^Ab^	4.21 ± 2.87 ^Bb^	80.43 ± 33.49 ^Aa^
	Yes (OA)	55.35 ± 28.78 ^Aa^	19.89 ± 4.01 ^Ab^	13.67 ± 16.42 ^ABb^	72.25 ± 18.03 ^Aa^
Young	No (Y)	25.45 ± 4.33 ^Aa^	12.87 ± 2.01 ^Bb^	24.50 ± 9.95 ^ABb^	112.09 ± 0.25 ^Aa^
	Yes (YA)	37.40 ± 17.34 ^Aa^	16.68 ± 1.89 ^ABb^	27.37 ± 23.45 ^Ab^	83.64 ± 25.10 ^Aa^

*Lower case letters (a, b) are used, for each group (age and use of alendronate), to indicate significant differences when comparing the conditions (fracture x no fracture) (*p* < 0.05). In each line, when the same lower case letter is used, the difference between the means is not statistically significant. Uppercase letters (A, B) are used, for each condition (fracture and no fracture), to indicate significant differences when comparing the groups (age and use of alendronate) (*p* < 0.05). In each column, when the same uppercase letter is used, the difference between the means is not statistically significant.*

Regarding the cross-section on the contralateral side, middle aged animals presented higher cross-sectional area (20.09 mm^2^) that young animals (15.09 mm^2^; *p* < 0.05). For the alendronate factor, there was no difference between treated (18.48 mm^2^) and untreated animals (17.64 mm^2^). Cross-sectional area values in old animals that were treated (19.89 mm^2^) or untreated (20.30 mm^2^) tended to be higher than those in young animals without the drug treatment (12.87 mm^2^). However, the factors alendronate and age/alendronate did not present a statistically significant difference (*p* > 0.05).

Regarding the flexural strength on the fracture side, young animals presented higher flexural strength value (26.17 MPa) than middle aged animals (8.94 MPa; *p* < 0.05). However, there were no differences between alendronate-treated (19.66 MPa) and untreated (11.45 MPa) animals. The factors alendronate and age/alendronate did not present a statistically significant difference (*p* > 0.05).

Analyzing the flexural strength on the non-fracture side, there was no difference between young and middle aged animals *p* > 0.05). For the alendronate administration, there was no difference between treated (77.23 MPa) and untreated animals (91.73 MPa). The factors alendronate and age/alendronate did not present a statistically significant difference (*p* > 0.05).

Table 4 demonstrates the cross-sectional area and flexural strength comparison between femurs within the same animal. The fracture side had higher values of cross-sectional area compared to the non-fracture side (*p* < 0.05). However, flexural strength, were higher on the values non-fractured side, whether with or without alendronate treatment (*p* < 0.05).

**TABLE 4:** Comparison of cross-section and flexural strength in animals with and without alendronate treatment between the femurs on the fractured and non-fractured side.

Age	Alendronate	Cross-section (mm2)–fracture Mean ± SD	Cross-section (mm2) no fracture Mean ± SD	*P* Value	Flexural strength (MPa)–fracture Mean ± SD	Flexural strength (MPa) no fracture Mean ± SD	*P* Value
Middle-age	No (O)	51.70 ± 22.37	20.30 ± 2.32	0.002	4.21 ± 2.87	80.43 ± 33.49	<0.001
	Yes (OA)	55.35 ± 28.78	19.89 ± 4.01	0.006	13.67 ± 16.42	72.25 ± 18.03	<0.001
Young	No (Y)	25.45 ± 4.33	12.87 ± 2.01	0.008	24.50 ± 9.95	112.09 ± 0.25	0.002
	Yes (YA)	37.40 ± 17.34	16.68 ± 1.89	0.022	27.37 ± 23.45	83,64 ± 25.10	0.006

*Paired *T* Test, with significance level 0.05.*

In flowchart 1, we summarize the main findings of the study.

**FLOWCHART 1:**
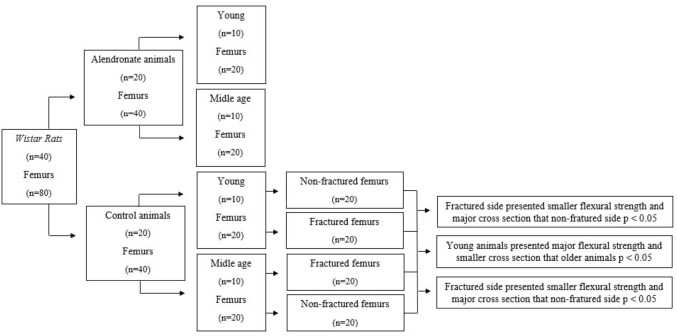
Main findings of the study.

**FIGURE 2:**
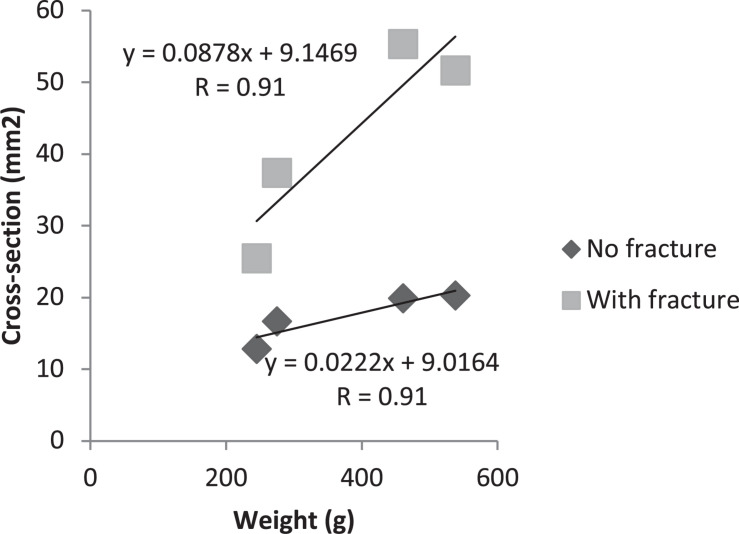
Person’s correlation test between weight and cross-section, with and without fracture.

**FIGURE 3:**
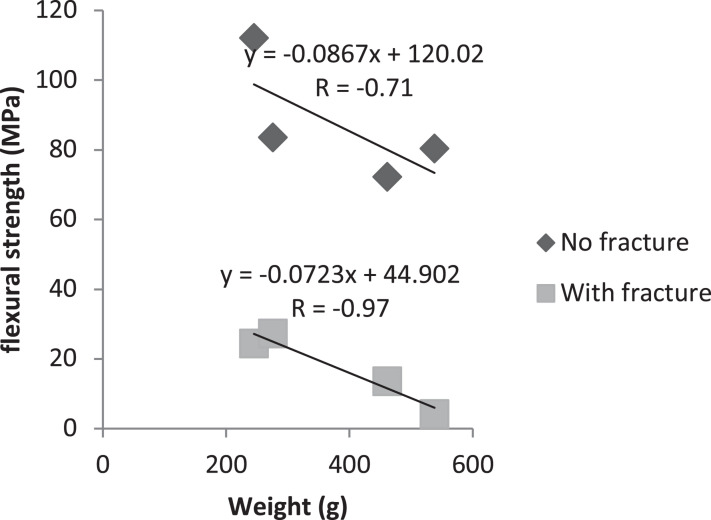
Person’s correlation test between weight and flexural strength, with and without fracture.

Correlations between weight and cross-section, and between weight and flexural strength were evaluated. Positive correlation was observed (*R* = +0.91) between cross-sectional area and weight, both on the fracture and contralateral sides (Figure 2). On the other hand, a negative correlation (*R* = −0.91 and *R* = −0.97) between flexural strength and weight, both for the fracture and contralateral sides (Figure 3).

## Discussion

This study examined the effect of aging on the healing of controlled femoral fracture, and whether alendronate treatment will play a role in modifying the effect of age. We found important findings related to age. Young animals presented lower cross-section than older animals. Besides, on fractured side, young animals presented major flexural strength than older animals. Related to the use of alendronate, there was no difference in relation to cross-section and flexural strength.

Cross-sectional area of bone was tested as a factor of change in animal size with age. We observed that Younger animals had a smaller cross-section than middle aged animals. According to the study by Kang et al. (2016), bone mineral density is widely used in the assessment of bone strength, but it is not enough to predict the risk of fractures in an individual. Studies have reported that bone geometry is one of the major determinants of the mechanical strength and flexural strength along the cortical surfaces of the bone (Faulkner et al., 2006; Kaptoge et al., 2008; LaCroix et al., 2010). Some factors, including age, have substantial effects on bone. The velocity of bone resorption exceeds bone apposition with aging. Besides that, the weight of the animals influenced in the cross-section, as shown in the study by Ong et al. (2014), which affirms that a greater weight is associated to a higher bone density and greater bone geometry, but this do not influence in a lower risk of fracture. This was demonstrated in the present study, where there was a positive correlation of the weight with the cross-section, showing that middle aged animals, with a higher weight (mean 499.16g) presented greater cross-section and young animals with, lower weight (mean 262.50g) presented lower cross-section.

Although the non-fracture side showed smaller cross-sectional area in young versus middle aged animals, there was no difference in flexural strength in any of the groups on that side. On the other hand, the fractured side showed a difference in flexural strength, where young animals presented greater resistance than middle aged animals. Regarding the fractured side, there was no difference in flexural strength between treated and untreated animals, whether old or young Beck et al. (2001), reported that lower weight may improve the bone strength though the mechanical tensions capable of stimulating the beneficial response of the bone. Greater weight can directly influence the bone through gravitational load (Zhao et al., 2007). In addition, other studies have shown that the negative impact of greater weight on the bone may be due to increase of parathyroid hormone concentration (Pitroda et al., 2009), reduction of inflammatory cytokines (Braun and Schett, 2012) and involvement of mesenchymal stem cells in osteoblastogenic cells (Ilich et al., 2014). This affirmation corroborates with the findings of this study, that there was a negative correlation of the weight with the resistance to flexion, in which, with the increase of the weight of the animal the resistance of flexion of the femur decreases.

The process of bone healing in young and old individuals work differently. In order to this process to occur, it is necessary to balance the reabsorption of the initial callus by osteoclasts and the deposition of lamellar bone by osteoblasts, which begins around three to 4 weeks after the trauma, both in animal models and humans. This process can take years to achieve a regenerated bone structure and can occur faster in young individuals because they present a more active metabolism (Wendeberg, 1961). In addition, according to Manalagas and Parfitt, in 2010, the aging process is related to hormonal changes, such as reduction of antioxidant capacity, sexual hormone deficiency and excess glucocorticoids, which may have influence in the healing process of tissues (Kaptoge et al., 2008).

Relate to age, it is known that in childhood and adolescence, bone formation overcomes bone resorption, reaching peak bone mass around 30 years of age. After this process, bone resorption slowly begins to overcome bone formation (Dean, 2003), in other words, aging is associated with an accelerated loss of bone mass. With this increase in bone resorption, serum calcium is reduced, and this explains the importance of correct nutrition and supplementation of calcium and vitamin D in the elderly (Bonjour et al., 2008; Reid et al., 2008). In addition to calcium, alkaline phosphatase is highly studied in research with postmenopausal women, in which the accelerated bone loss process occurs, and the serum level of alkaline phosphatase increases as a result of bone remodeling (Bhattarai et al., 2014; LeKhi et al., 2012). For these reasons, we tested serum levels of calcium and alkaline phosphatase, knowing that both markers act in the process of bone formation and remodeling.

Authors, in 2015, reported that serum calcium slightly decreased during treatment with alendronate, due to a reduction in the flow of calcium from the bone, causing an increase in parathyroid secretion, which increases bone turnover (Sakai et al., 2015). Therefore, supplementation of vitamin D and calcium are indicated during treatment with alendronate, in order to maintain the right balance of calcium levels. Our results did not corroborate with the findings of previous studies, in which the serum calcium level remained stable in the groups treated with bisphosphonate. This may be because the biochemical test was performed with only 20% of the sample, which may have weakened the statistical power of this analysis. Regarding alkaline phosphatase, there was no statistical difference between the groups. Goes et al., in 2012 evaluated the effect of alendronate on serum alkaline phosphatase levels and loss of periodontal bone in Wistar rats. The authors concluded that the induction of periodontitis caused reduction of levels of alkaline phosphatase and severe alveolar bone loss, revealing that the serum alkaline phosphatase levels reduces with bone loss (Goes et al., 2012).

In the present study, animals treated with alendronate, middle aged or young, showed no difference in flexural strength. Researchers, in 2006, described that increases related to fracture resistance are observed after weeks or months of alendronate administration. In their studies, treatment with alendronate demonstrated increased density of bone mineral, cross-sectional area of the bone, and mechanical strength of the femur (Spadaro et al., 2006). Our results do not corroborate with these findings. It is possible that our sample was not enough to detect the significance (Type II error). It is worth mentioning that in the data analysis, we observed higher values of flexural strength in the groups treated with alendronate than in control group.

There were several limitations during the execution of this work, since the experimental design included middle aged animals. In order to perform the research, in the group with older animals, it was necessary to wait 18 months after their birth to perform the surgical procedures, that which makes the study quite long. Other point related to age is the chose for groups. In a future study, we suggest adding more age groups to analyze the influence of age throughout life. Another point worth mentioning was the use of stable internal fixation with plates and screws. In the previous work of our team (Weiss et al., 2018), we used the fixation only in young animals. With middle aged animals, often fractures of the femur occurred during the drilling of screws, because of the mineral bone density.

Other important limitation of the study is the absence of imaginological exams, like X-Ray or Micro-UT before mechanical tests. Future studies should add these exams in order to help elucidate the bone repair process in view of the age and the presence of alendronate.

In short, there was no difference of calcium and alkaline phosphatase in the study. Age has influence in cross-section and flexural strength during the bone repair process of middle aged or young rats. It was shown to negatively influence of age in flexural strength of femoral fracture callus. Smaller cross-section in young rats was associated with greater flexural strength. Finally alendronate showed no association with these factors.

## Data Availability Statement

The original contributions presented in the study are included in the article/supplementary material, further inquiries can be directed to the corresponding author/s.

## Ethics Statement

The animal study was reviewed and approved by Ethics Committee on the Use of Animals (ECUA 393), Positivo University.

## Author Contributions

LB designed the study and prepared the first draft of the manuscript. She is guarantor. Authors PC, GK, JG, LC, ME, and JZ contributed to the experimental work and writing the manuscript. CG was responsible for statistical analysis and contribute writing the manuscript. RS designed the study, performed some surgical procedures and coordinated the research. All authors read and approved the final version of the article.

## Conflict of Interest

The authors declare that the research was conducted in the absence of any commercial or financial relationships that could be construed as a potential conflict of interest.
